# Comparative Characteristics of the Metabolic Syndrome Prevalence in Patients With Schizophrenia in Three Western Siberia Psychiatric Hospitals

**DOI:** 10.3389/fpsyt.2021.661174

**Published:** 2021-07-02

**Authors:** Elena G. Kornetova, Alexander N. Kornetov, Irina A. Mednova, Anastasia A. Goncharova, Valeria I. Gerasimova, Ivan V. Pozhidaev, Anastasiia S. Boiko, Arkadiy V. Semke, Anton J. M. Loonen, Nikolay A. Bokhan, Svetlana A. Ivanova

**Affiliations:** ^1^Endogenous Disorders Department, Mental Health Research Institute, Tomsk National Research Medical Center of the Russian Academy of Sciences, Tomsk, Russia; ^2^University Hospital, Siberian State Medical University, Tomsk, Russia; ^3^Fundamental Psychology and Behavioral Medicine Department, Siberian State Medical University, Tomsk, Russia; ^4^Molecular Genetics and Biochemistry Laboratory, Mental Health Research Institute, Tomsk National Research Medical Center of the Russian Academy of Sciences, Tomsk, Russia; ^5^PharmacoTherapy, -Epidemiology and -Economics, Groningen Research Institute of Pharmacy, University of Groningen, Groningen, Netherlands; ^6^Addictive Disorders Department, Mental Health Research Institute, Tomsk National Research Medical Center of the Russian Academy of Sciences, Tomsk, Russia; ^7^Psychiatry, Addiction Psychiatry and Psychotherapy Department, Siberian State Medical University, Tomsk, Russia

**Keywords:** schizophrenia, psychosis, antipsychotics, metabolic syndrome, pathology

## Abstract

**Objective:** The purpose of this study was to compare the prevalence of MetS and the associated sociodemographic, clinical, and pharmacotherapeutic characteristics of patients with schizophrenia in three psychiatric hospitals in the West Siberian region.

**Methods:** Patients with a clinical diagnosis of schizophrenia (ICD-10: F20) and an age between 18 and 60 years were included in the study after giving informed consent. Metabolic syndrome was diagnosed according to the International Diabetes Federation criteria. This research was carried out at three Western Siberian psychiatric hospitals in Kemerovo, Tomsk, and Omsk. The study population included respectively 94, 131, and 91 inpatients with schizophrenia. We carried out schizophrenia symptoms assessment by PANSS, antipsychotic therapy evaluation, anthropometry, and biochemical analysis. Statistical Analysis included the Shapiro–Wilk test, non-parametric Kruskal–Wallis *H*-test for independent samples, Mann–Whitney *U*-test for independent samples, the chi-square test, stepwise multiple regression analyses. The level of significance was *p* < 0.05.

**Results:** The metabolic syndrome prevalence was higher among patients in Tomsk (36.6%), compared with Kemerovo (20.2%, *p* = 0.008) or Omsk (18.7%, *p* = 0.004), mainly due to the high prevalence of abdominal obesity, while men from Tomsk were more susceptible to this condition than men from other regions (*p* < 0.05). Patients from Omsk had the highest severity schizophrenia symptoms according to PANSS, and patients from Tomsk had the lowest severity of positive symptoms according to PANSS. Patients from Tomsk had the minimum duration of antipsychotic therapy compared with the patient from Kemerovo (*p* = 0.017) and from Omsk (*p* = 0.000019), but most patients from Tomsk received second-generation atypical antipsychotics, while patients from Omsk received mainly conventional antipsychotics (*p* = 0.0001). Multiple regression analysis showed that metabolic syndrome associated with schizophrenia duration and body mass index, although the association was not so strong (adjusted *R*^2^ = 0.2435, *p* < 0.0001).

**Discussion:** The study illustrates that in different psychiatric hospitals within the same region, the prevalence of metabolic syndrome in patients with schizophrenia can vary significantly, which dictates the need to look for opportunities to minimize the risk of its occurrence, taking into account the experience of each hospital.

## Introduction

Schizophrenia is a chronic and severe mental disorder. There is generally accepted that its prevalence in the population is relatively stable at 1% (1).

Schizophrenia associated with a high global disease burden (2) and a decreased life expectancy of 11–20 years compared to the general population (3). One of the most common causes of death in patients with schizophrenia is cardiovascular diseases (4), closely associated with the presence of metabolic syndrome (MetS), which prevalence in patients with schizophrenia is significantly higher than in the general population (5). MetS in patients with schizophrenia is a complex phenomenon, which is partly due to the disorder itself, and partly due to adverse events of antipsychotic treatment (6, 7).

There is some evidence that the prevalence of the MetS is heterogeneous depending on the region of the world and even region of the country, and the prevalence of its cluster components has some associations with cross-culturally and cross-ethnicity (8–10). Also, the contribution of certain socioeconomic factors to the prevalence of the MetS is discussed: its components were much less frequent among individuals with a higher educational level, higher income, and occupational status, and those having a life partner (11). The prevalence of MetS among patients with schizophrenia was 23.9% in a meta-analysis conducted by Iranian researchers. The prevalence was higher among women than among men (34 vs. 10.8%). There is noteworthy that no relationship between the prevalence of MetS and age or duration of illness (12). A study from Turkey showed a higher prevalence of MetS among patients with schizophrenia and first-episode psychosis compared with the randomly selected healthy volunteers, regardless of the MetS criteria used. The MetS patients had a higher mean of age, a longer duration of disease and treatment compared to patients without MetS (13). Similar results were found in a study in Palestine, there were also discovered that systolic blood pressure, high triglycerides, high fasting plasma glucose, and low high-density lipoprotein cholesterol (HDL-C) were significant predictors of MetS (14). Spanish researchers, in addition to assessing the prevalence of MetS, also found that patients with schizophrenia who met the remission criteria were younger and had a lower body mass index (BMI) (15). In a Singapore study, the prevalence of MetS in patients with schizophrenia was 46.0% (16). Thus, the frequency of MetS and its components in patients with schizophrenia is not constant and depends on many factors that should be studied comprehensively.

The described differences in the epidemiology of both schizophrenia and MetS indicate the importance of comparative population and subpopulation studies to identify potential risk groups. While wide variations have been reported little is known about the factors underlying and the importance of understanding them cannot be overemphasized. A large meta-analysis by Indian researchers showed a similar to European patients' prevalence of MetS (29.83%) in persons with schizophrenia (17). Another study reported a lower prevalence of MetS in drug-naive patients with schizophrenia than in the general population (18).

Researchers from Japan found a greater prevalence of MetS in the outpatient group compared with the inpatient group with schizophrenia. This difference can be explained by the peculiarities of the Japanese mental health care system, which provides for constant lifestyle management in hospitals (19, 20). Similar data were obtained in Indonesia: MetS was even less common in inpatients with schizophrenia than in the general population, regardless of antipsychotic treatment (21). The authors explain these differences by the financial burden of patients and the inability to buy extra food during hospitalization.

Separate studies conducted in Korea and Taiwan compare a cohort of patients with schizophrenia with the general population, revealing a high prevalence of MetS in the first group, which is consistent with data from European studies (22, 23). However, to date, few comparisons have been made between individual subpopulations of patients with schizophrenia. Among them, we can highlight the study of differences in the prevalence and characteristics of MetS in outpatients with schizophrenia in different countries (19), or living in communities (24). In several studies, MetS and its components were compared in schizophrenia patients with mood disorders (25, 26). There are practically no comparative studies of MetS in schizophrenia inpatients in different psychiatric hospitals, geographically located within the same region, which allow one to assess the role of antipsychotic pharmacotherapeutic schemes and combinations characteristic of individual hospitals in the development of MetS.

The question of the effect of antipsychotic treatment on MetS in patients with schizophrenia is remaining open. A major recent review noted differences between antipsychotics in terms of metabolic side-effects, with olanzapine and clozapine exhibiting the worst profiles and aripiprazole, brexpiprazole, cariprazine, lurasidone, and ziprasidone the most benign profiles. The risk factors of antipsychotic-induced metabolic change were increased baseline weight, male sex, and non-white ethnicity (27). At the same time, much less data exist on the effect of antipsychotic polypharmacy on the prevalence of MetS. There was also shown a possible protective effect of drug combinations including aripiprazole for diabetes and hyperlipidemia, compared to other combinations and/or monotherapy (28). Other authors report that the prevalence of MetS does not differ in schizophrenia whether patients are receiving polypharmacy and monotherapy (29). In a study comparing polypharmacy and clozapine monotherapy, clozapine had a more significant effect on the development of MetS (28). There is also evidence that antipsychotic polypharmacy can increase the risk of the pre-metabolic syndrome, even after adjusting for patients' lifestyle characteristics (30, 31). We have not come across studies comparing the prevalence of MetS and its components in inpatients with schizophrenia in hospitals within the same region, for example, a country or area. This formulation of the question can reveal subjective differences, for example, the approaches to treatment adopted in a particular hospital.

Thus, despite the existence of differences between subpopulations of patients with schizophrenia, the amount of data on this topic remains insufficient, which determines the relevance of this study. The purpose of this study was to compare the prevalence of MetS and the associated sociodemographic, clinical, and pharmacotherapeutic characteristics of patients with schizophrenia in three psychiatric hospitals in the West Siberian region. We hypothesized that there may be differences in MetS prevalence in patients with schizophrenia taking antipsychotic therapy across multiple psychiatric hospitals within the same region, associated with differing sociodemographic, clinical, and pharmacotherapeutic characteristics. Western Siberia is an industrial and scientific region with a continental climate, long cold winters, short summers, located in the geographic center of Russia, known for its hydrocarbon deposits and an extensive network of gas and oil production. The administrative centers are large cities, but the region has a relatively low population density, due to which psychiatric hospitals are concentrated in large cities. Mental health care in this region, as well as throughout Russia, is funded by the state and is free of charge for patients. Hospitals, as a rule, do not have significant differences among themselves in the organization of mental health care.

## Materials and Methods

### Study Population

This research was carried out from September to December 2019 at three Western Siberian psychiatric hospitals in Kemerovo, Tomsk, and Omsk. The study population included respectively 94, 131, and 91 inpatients with schizophrenia who written informed consent and met the following inclusion criteria:

- presence of schizophrenia diagnosed according to the International Statistical Classification of Diseases and Related Health Problems, 10th Revision (ICD-10: F20);
- antipsychotics therapy for at least 6 months before entering the study;
- age range 18–60 years.

Exclusion criteria:

- a history of eating disorders;
- a history of alcohol or drug abuse;
- diabetes;
- treatment for organic comorbidities.

The groups of patients recruited in each of the hospitals did not differ in sex, age, educational level, and marital status (Table 1). All patients received therapy with conventional or/and second-generation atypical antipsychotics.

**Table 1:** Study population characteristics.

**Parameter**	**Psychiatric hospital patients**, ***n***			
	**(1) Kemerovo, 94**	**(2) Tomsk, 131**	**(3) Omsk, 91**	***p*-value**
**Age and sex**				
Age, Me [Q1; Q3] years	34.5 [29; 40]	32 [27; 40]	34 [30; 42]	0.0640
Sex [male, *n* (%)/female, *n* (%)]	48 (51.1)/46 (48.9)	69 (52.7)/62 (47.3)	50 (54.9)/41 (45.1)	0.86836
**Education level**, ***n*** **(%)**				
Higher	20 (21.3%)	46 (35.1%)	9 (9.9%)	0.112
Incomplete higher	10 (10.6%)	20 (15.3%)	6 (6.6%)	
Secondary	61 (64.9%)	61 (46.5%)	63 (69.2%)	
Incomplete secondary	3 (3.2%)	4 (3.1%)	13 (14.3%)	
**Family status**, ***n*** **(%)**				
Married	5 (5.3%)	19 (14.5%)	14 (15.4%)	0.219
Singles	75 (79.8%)	94 (71.8%)	65 (71.4%)	
Divorced	14 (14.9%)	18 (13.7%)	12 (13.2%)	

*Comparisons between groups were performed using Kruskal–Wallis test for age and chi-squared test for the rest of the variables; Me [Q1; Q3], median and quartiles (first and third)*.

### Ethics Approval Statement

The study was conducted according to the guidelines of the Declaration of Helsinki, and approved by the Ethics Committee of Mental Health Research Institute of the Tomsk National Research Medical Center of the Russian Academy of Sciences (protocol number 187, date of approval 2018-04-24). All participants provided written informed consent to participate in this study.

### Schizophrenia Criteria and Symptom Severity Assessment

We used the World Health Organization World Mental Health Composite International Diagnostic Interview (WHO WMH-CIDI) for schizophrenia diagnostics. Symptom severity assessment was carried out with the Positive and Negative Syndrome Scale (PANSS) (32). Information about work capacity or disability as a result of mental disorder, duration of the disorder, and duration of antipsychotic use were taken from available medical records. The doses of antipsychotics that patients received were calculated to uniformity for chlorpromazine equivalent (CPZeq) (33).

### Definition of MetS

We used the criteria of the IDF for MetS diagnostics (34). These criteria require that MetS is diagnosed in a patient with central obesity (WC more than 94 cm in men and more than 80 cm in women) and the presence of any two of the following four signs:

- the concentration of triglycerides in serum is higher than 1.7 mmol/L (150 mg/dl) or lipid-lowering therapy is carried out;
- the concentration of high-density lipoprotein in serum is below 1.03 mmol/L (40 mg/dl) in men and 1.29 mmol/L (50 mg/dl) in women;
- the arterial blood pressure level is systolic above 130 mmHg or diastolic above 85 mmHg (or with treatment of previously diagnosed hypertension);
- serum glucose concentration is >5.6 mmol/L (100 mg/dl; or previously diagnosed type 2 diabetes).

### Blood Sampling and Biochemical Parameters

After 12 h of overnight fasting, blood samples were taken into BD Vacutainer® with a clot activator (BD, Franklin Lakes, NJ, USA) for biochemical tests of glucose, HDL-C, and triglycerides using commercial kits (Cormay, Łomianki, Poland).

### Statistical Analysis

We used the Shapiro–Wilk test to assess the data correspondence to the normal distribution at the first stage. The significance of intergroup differences was evaluated using the non-parametric Kruskal–Wallis *H*-test for independent samples. The pairwise analysis was performed using Mann–Whitney *U*-test for independent samples. The chi-square (χ^2^) test was used to analyze the categorical variables. Bonferroni correction was applied for multiple comparisons to erroneous inferences prevent. Descriptive statistics are presented as the median with 25 and 75% quartiles, Me (Q1; Q3). We conducted stepwise multiple regression analyses to examine the relevance of variables for the presence of the MetS. We entered into the model variables that in univariate analyses were different between patients in three groups (psychiatric hospitals in Kemerovo, Tomsk, and Omsk). The level of significance was *p* < 0.05.

## Results

### Employment Status of the Study Population

More patients from Tomsk had a job compared to a patient from Kemerovo and Omsk (*p* = 0.0405 and 0.0019, respectively). There were fewer unemployed patients from Omsk compared to patients from Kemerovo and Tomsk (*p* = 0.0103 and 0.0099, respectively). However, the majority of patients from Omsk had psychiatric disability compared to patients from Kemerovo and Tomsk (*p* = 0.031 and 0.000, respectively). A psychiatric disability was also more common in Kemerovo than in Tomsk patients (*p* = 0.0266; Table 2).

**Table 2:** Employment status of the study population, *n* (%).

**Employment**	**Psychiatric hospital patients**, ***n***			
	**(1) Kemerovo, 94**	**(2) Tomsk, 131**	**(3) Omsk, 91**	***p*-value**
Working	13 (13.8%)	30 (22.9%)	8 (8.8%)	*p*_1−2_ = 0.0405^*^
				*p*_1−3_ = 0.138
				*p*_2−3_ = 0.0019^*^
Unemployed	25 (26.6%)	40 (30.5%)	12 (13.2%)	*p*_1−2_ = 0.259
				*p*_1−3_ = 0.0103^*^
				*p*_2−3_ = 0.0009^*^
Psychiatric disability	56 (59.6%)	61 (46.6%)	71 (78.0%)	*p*_1−2_ = 0.0266^*^
				*p*_1−3_ = 0.0031^*^
				*p*_2−3_ = 0.000^*^

* *p < 0.05, statistically significant difference; comparisons between groups were performed using the chi-squared test*.

### Schizophrenia Clinical Characteristics Comparison

Patients from Omsk had a longer disease duration than patients from Tomsk (*p* = 0.032) and Kemerovo (*p* = 0.075). Patients from Tomsk had a minimum score of PANSS positive symptoms compared with the patient from Kemerovo (*p* = 0.00001) and from Omsk (*p* = 0.00000). The maximum total PANSS score was in patients from Omsk (*p* = 0.035 and 0.001 compared to patients from Kemerovo and Tomsk, respectively), mainly due to negative symptoms (*p* = 0.002 compared to patients from Kemerovo; Table 3). The minimum PANSS positive symptoms score was recorded among males from Tomsk, as compared to males from Kemerovo (*p* = 0.0029) and from Omsk (*p* = 0.00007). The PANSS total score was higher in males from Omsk than in males from Tomsk (*p* = 0.0346; Table 4). The minimum positive PANSS symptoms score was recorded in females from Tomsk compared with females from Kemerovo (*p* = 0.0046) and from Omsk (*p* = 0.00057). Females from Omsk had a higher PANSS negative symptoms score than females from Kemerovo (*p* = 0.0176), as well as a higher total PANSS score than females from Tomsk (*p* = 0.047; Table 5).

**Table 3:** Comparative characteristics and evaluation of clinical parameters and antipsychotic therapy in the study population (Me [Q1; Q3]).

**Parameter**	**Psychiatric hospital patients**, ***n***			
	**(1) Kemerovo, 94**	**(2) Tomsk, 131**	**(3) Omsk, 91**	***p*-value**
**Clinical parameters**				
Schizophrenia onset age	23 [19; 29]	23 [19; 28]	23 [19; 29]	0.5934
Duration of disease, years	8.5 [5; 15]	9 [3; 16]	12 [8; 16]	*p*_1−2_ = 0.776
				*p*_1−3_ = 0.075
				*p*_2−3_ = 0.032^*^
PANSS positive symptoms score	24 [20; 26]	20 [15; 24]	25 [20; 28]	*p*_1−2_ = 0.00001^*^
				*p*_1−3_ = 0.963
				*p*_2−3_ = 0.00000^*^
PANSS negative symptoms score	23.5 [20; 26]	25 [21; 28]	26 [23; 28]	*p*_1−2_ = 0.126
				*p*_1−3_ = 0.002^*^
				*p*_2−3_ = 0.312
PANSS general psychopathology symptoms score	52 [48; 56]	52 [44; 59]	56 [45; 62]	*p*_1−2_ = 0.881
				*p*_1−3_ = 0.077
				*p*_2−3_ = 0.051
PANSS total score	99 [91; 105]	97 [86; 108]	108 [91; 116]	*p*_1−2_ = 0.368
				*p*_1−3_ = 0.035^*^
				*p*_2−3_ = 0.001^*^
**Antipsychotic therapy characteristics**				
Antipsychotic therapy duration, years	7.5 [3; 12]	4 [1; 9]	9 [3; 15]	*p*_1−2_ = 0.017^*^
				*p*_1−3_ = 0.908
				*p*_2−3_ = 0.000019^*^
Total antipsychotic dose, CPZeq	450 [250; 750]	350 [200; 598.5]	435 [275; 798]	0.086

* *p < 0.05, statistically significant difference; comparisons between groups were performed using the Kruskal–Wallis H-test; pairwise analysis was performed using Mann–Whitney U-test for independent samples with Bonferroni correction for multiple comparisons for the rest of the variables; Me [Q1; Q3], median and quartiles (first and third); PANSS, positive and negative syndrome scale*.

**Table 4:** Comparative characteristics and evaluation of clinical parameters and antipsychotic therapy in the male part of the study population (Me [Q1; Q3]).

**Parameter**	**Psychiatric hospital patients**, ***n***			
	**(1) Kemerovo, 48**	**(2) Tomsk, 69**	**(3) Omsk, 50**	***p*-value**
**Clinical parameters**				
Schizophrenia onset age	22 [19; 24.5]	22 [19; 26]	21 [19; 27]	0.922
Duration of disease, years	7.5 [5; 15]	8 [3; 16]	12 [8; 16]	*p*_1−2_ = 0.9801
				*p*_1−3_ = 0.09
				*p*_2−3_ = 0.076
PANSS positive symptoms score	23 [19.5; 26.5]	19 [15; 24]	25 [19; 28]	*p*_1−2_ = 0.0029^*^
				*p*_1−3_ = 0.292
				*p*_2−3_ = 0.00007^*^
PANSS negative symptoms score	24 [21.5; 27]	25 [21; 30]	27 [23; 29]	0.119
PANSS general psychopathology symptoms score	52 [49; 56]	52 [44; 57]	57 [45; 62]	0.169
PANSS total score	99.5 [93; 105]	97 [85; 109]	108 [88; 116]	*p*_1−2_ = 0.405
				*p*_1−3_ = 0.361
				*p*_2−3_ = 0.0346^*^
**Antipsychotic therapy characteristics**				
Antipsychotic therapy duration, years	8.5 [5; 15]	4 [2; 9]	8.5 [4; 15]	*p*_1−2_ = 0.00206^*^
				*p*_1−3_ = 0.886
				*p*_2−3_ = 0.0032^*^
Total antipsychotic dose, CPZeq	446.25 [250; 750]	350 [200; 587.5]	500 [300; 782]	0.071

* *p < 0.05, statistically significant difference; comparisons between groups were performed using the Kruskal–Wallis H-test; pairwise analysis was performed using Mann–Whitney U-test for independent samples with Bonferroni correction for multiple comparisons for the rest of the variables; Me [Q1; Q3], median and quartiles (first and third); PANSS, positive and negative syndrome scale*.

**Table 5:** Comparative characteristics and evaluation of clinical parameters and antipsychotic therapy in the female part of the study population (Me [Q1; Q3]).

**Parameter**	**Psychiatric hospital patients**, ***n***			
	**(1) Kemerovo, 46**	**(2) Tomsk, 62**	**(3) Omsk, 41**	***p*-value**
**Clinical parameters**				
Schizophrenia onset age	25.5 [21; 32]	23.5 [19; 30]	24 [20; 31]	0.418
Duration of disease, years	9.5 [5; 16]	10 [4; 16]	11 [8; 15]	0.390
PANSS positive symptoms score	24 [21; 26]	20 [14; 24]	24 [21; 28]	*p*_1−2_ = 0.0046^*^
				*p*_1−3_ = 0.488
				*p*_2−3_ = 0.00057^*^
PANSS negative symptoms score	22 [20; 25]	24.5 [20; 27]	26 [24; 28]	*p*_1−2_ = 0.279
				*p*_1−3_ = 0.0176^*^
				*p*_2−3_ = 0.565
PANSS general psychopathology symptoms score	51.5 [48; 55]	52 [45; 60]	56 [51; 59]	0.120
PANSS total score	99 [90; 105]	97 [86; 107]	107 [95; 113]	*p*_1−2_ = 0.656
				*p*_1−3_ = 0.131
				*p*_2−3_ = 0.047^*^
**Antipsychotic therapy characteristics**				
Antipsychotic therapy duration, years	5 [2; 11]	3 [0.6; 8]	10 [2; 13]	*p*_1−2_ = 0.317
				*p*_1−3_ = 0.434
				*p*_2−3_ = 0.0054^*^
Total antipsychotic dose, CPZeq	450 [250; 717]	316.4 [200; 598.5]	400 [226.5; 798]	0.313

* *p < 0.05, statistically significant difference; comparisons between groups were performed using the Kruskal–Wallis H-test; pairwise analysis was performed using Mann–Whitney U-test for independent samples with Bonferroni correction for multiple comparisons for the rest of the variables; Me [Q1; Q3], median and quartiles (first and third); PANSS, positive and negative syndrome scale*.

### Comparison of Antipsychotic Therapy

Patients from Tomsk had the minimum duration of antipsychotic therapy (Table 3) compared with the patient from Kemerovo (*p* = 0.017) and from Omsk (*p* = 0.000019). The minimum duration of antipsychotic therapy was recorded in males from Tomsk as compared to males from Kemerovo (*p* = 0.00206) and from Omsk (*p* = 0.0032; Table 4). Females from Omsk had a longer duration of antipsychotic therapy than females from Tomsk (*p* = 0.0054; Table 5). Most patients from Tomsk received atypical antipsychotics, while patients from Omsk received mainly conventional antipsychotics (*p* = 0.0001; Table 6). The majority of patients from Tomsk took antipsychotic monotherapy as compared with patients from Kemerovo and Omsk (*p* = 0.0186 and 0.001, respectively). Patients from Omsk most often received a combination of two antipsychotics compared to patients from Kemerovo and Tomsk (*p* = 0.046 and 0.0019, respectively). The patients from Kemerovo were more likely to take three antipsychotics at the same time as compared with the patients from Tomsk (*p* = 0.0224; Table 6).

**Table 6:** Comparative characteristics and evaluation of antipsychotics generations and antipsychotics combinations, *n* (%).

**Antipsychotics combinations**	**Psychiatric hospital patients, n**			
	**(1) Kemerovo, 94**	**(2) Tomsk, 131**	**(3) Omsk, 91**	***p*-value**
**Antipsychotics generations**				
Conventional antipsychotics	53 (56.4%)	22 (16.8%)	75 (82.4%)	*p*_1−2_ = 0.0001^*^
Atypical antipsychotics	41 (43.6%)	109 (83.2%)	16 (17.6%)	*p*_1−3_ = 0.0001^*^
				*p*_2−3_ = 0.0001^*^
**Antipsychotics combinations**				
Monotherapy	51 (54.3%)	89 (67.9%)	*43 (50.0%)*	*p*_1−2_ = 0.0186^*^
				*p*_1−3_ = 0.1702
				*p*_2−3_ = 0.001^*^
Two antipsychotics combine	33 (35.1%)	37 (28.3%)	43 (50.0%)	*p*_1−2_ = 0.1373
				*p*_1−3_ = 0.046^*^
				*p*_2−3_ = 0.0019^*^
Three antipsychotics combine	10 (10.6%)	5 (3.8%)	5 (5.4%)	*p*_1−2_ = 0.0224^*^
				*p*_1−3_ = 0.096
				*p*_2−3_ = 0.279

* *p < 0.05, statistically significant difference; comparisons between groups were performed using the chi-squared test*.

### Anthropometric Parameters

Patients from Tomsk, both in the general group (Table 7) and when divided by sex (Tables 8, 9), were characterized by a larger waist circumference and BMI than patients from Kemerovo or Omsk (*p* < 0.05 in each case).

**Table 7:** Comparative characteristics and evaluation of anthropometric and laboratory parameters in the study population (Me [Q1; Q3]).

**Parameter**	**Psychiatric hospital patients**, ***n***			
	**(1) Kemerovo, 94**	**(2) Tomsk, 131**	**(3) Omsk, 91**	***p*-value**
**Anthropometric and laboratory parameters**				
Waist circumference, cm	84.00 [75.75; 94.25]	95.00 [82.00; 105.50]	84 [78.00; 101.10]	*p*_1−2_ = 0.0001^*^
				*p*_1−3_ = 0.936
				*p*_2−3_ = 0.0001^*^
BMI, kg/m^2^	24.05 [22.10; 28.15]	27.00 [23.20; 33.30]	24.00 [21.40; 26.40]	*p*_1−2_ = 0.001^*^
				*p*_1−3_ = 0.448
				*p*_2−3_ = 0.0001^*^
**Laboratory parameters**				
Fasting glucose (mg/dl)	5.10 [4.80; 5.50]	4.90 [4.50; 5.30]	4.55 [4.17; 5.07]	*p*_1−2_ = 0.002^*^
				*p*_1−3_ = 0.0001^*^
				*p*_2−3_ = 0.007^*^
Triglyceride (mg/dl)	1.29 [1.01; 1.82]	1.40 [1.00; 1.90]	1.18 [0.80; 1.68]	*p*_1−2_ = 0.460
				*p*_1−3_ = 0.109
				*p*_2−3_ = 0.020^*^
HDL-C (mg/dl)	0.90 [0.72; 1.12]	0.94 [0.80; 1.20]	1.08 [0.84; 1.30]	*p*_1−2_ = 0.349
				*p*_1−3_ = 0.006^*^
				*p*_2−3_ = 0.057

* *p < 0.05, statistically significant difference; these data were analyzed using Kruskal–Wallis H-test; pairwise analysis was performed using Mann–Whitney U-test for independent samples with Bonferroni correction for multiple comparisons for the rest of the variables; Me [Q1; Q3], median and quartiles (first and third); BMI, body mass index; HDL-C, high-density lipoprotein cholesterol*.

**Table 8:** Comparative characteristics and evaluation of anthropometric and laboratory parameters in the male part of the study population (Me [Q1; Q3]).

**Parameter**	**Psychiatric hospital patients**, ***n***			
	**(1) Kemerovo, 48**	**(2) Tomsk, 69**	**(3) Omsk, 50**	***p*-value**
**Anthropometric and laboratory parameters**				
Waist circumference, cm	87.00 [80.00; 95.00]	97.00 [85.00; 105.00]	84.00 [80.00; 91.00]	*p*_1−2_ = 0.008^*^
				*p*_1−3_ = 0.443
				*p*_2−3_ = 0.001^*^
BMI, kg/m^2^	23.65 [22.10; 27.90]	26.90 [22.90; 31.60]	22.50 [21.10; 25.30]	*p*_1−2_ = 0.019^*^
				*p*_1−3_ = 0.215
				*p*_2−3_ = 0.0001^*^
**Laboratory parameters**				
Fasting glucose (mg/dl)	5.15 [4.80; 5.50]	4.90 [4.50; 5.30]	4.40 [4.09; 5.21]	*p*_1−2_ = 0.062
				*p*_1−3_ = 0.0001^*^
				*p*_2−3_ = 0.039^*^
Triglyceride (mg/dl)	1.30 [1.02; 1.80]	1.40 [0.90; 2.10]	1.22 [0.80; 1.77]	0.338
HDL-C (mg/dl)	0.90 [0.72; 1.11]	0.81 [0.70; 1.24]	0.98 [0.79; 1.24]	*p*_1−2_ = 0.327
				*p*_1−3_ = 0.314
				*p*_2−3_ = 0.042^*^

* *p < 0.05, statistically significant difference; these data were analyzed using Kruskal–Wallis H-test; pairwise analysis was performed using Mann–Whitney U-test for independent samples with Bonferroni correction for multiple comparisons for the rest of the variables; Me [Q1; Q3], median and quartiles (first and third); BMI, body mass index; HDL-C, high-density lipoprotein cholesterol*.

**Table 9:** Comparative characteristics and evaluation of anthropometric and laboratory parameters in the female part of the study population (Me [Q1; Q3]).

**Parameter**	**Psychiatric hospital patients**, ***n***			
	**(1) Kemerovo, 46**	**(2) Tomsk, 62**	**(3) Omsk, 41**	***p*-value**
**Anthropometric and laboratory parameters**				
Waist circumference, cm	80.50 [71.00; 94.00]	88.50 [82.00; 106.00]	84.00 [72.25; 93.00]	*p*_1−2_ = 0.004^*^
				*p*_1−3_ = 0.459
				*p*_2−3_ = 0.032^*^
BMI, kg/m^2^	24.30 [22.00; 30.15]	28.15 [23.20; 34.00]	25.00 [22.55; 28.60]	*p*_1−2_ = 0.024^*^
				*p*_1−3_ = 0.770
				*p*_2−3_ = 0.048^*^
**Laboratory parameters**				
Fasting glucose (mg/dl)	5.10 [4.84; 5.62]	4.80 [4.49; 5.25]	4.60 [4.20; 4.97]	*p*_1−2_ = 0.008^*^
				*p*_1−3_ = 0.0001^*^
				*p*_2−3_ = 0.096
Triglyceride (mg/dl)	1.29 [0.97; 1.93]	1.33 [1.10; 1.77]	1.15 [0.77; 1.58]	*p*_1−2_ = 0.724
				*p*_1−3_ = 0.165
				*p*_2−3_ = 0.040^*^
HDL-C (mg/dl)	0.91 [0.72; 1.16]	1.10 [0.90; 1.35]	1.16 [1.00; 1.36]	*p*_1−2_ = 0.013^*^
				*p*_1−3_ = 0.002^*^
				*p*_2−3_ = 0.484

* *p < 0.05, statistically significant difference; these data were analyzed using Kruskal–Wallis H-test; pairwise analysis was performed using Mann–Whitney U-test for independent samples with Bonferroni correction for multiple comparisons for the rest of the variables; Me [Q1; Q3], median and quartiles (first and third); BMI, body mass index; HDL-C, high-density lipoprotein cholesterol*.

### Laboratory Parameters

The maximum level of fasting glucose in the blood serum was registered in patients from Kemerovo (Table 7), the minimum—from Omsk (*p* < 0.05 in each case). Moreover, the maximum and minimum fasting glucose values were achieved mainly due to the level of this indicator in females (Table 9) and males (Table 8), respectively (*p* < 0.05 in each case). The triglyceride level was higher in patients from Tomsk (Table 7), mainly females (Table 9; *p* = 0.040) than from Omsk (*p* = 0.020). Females from Kemerovo had a lower HDL-C (Table 9) level than females from other regions (*p* < 0.05 in each case).

### Prevalence of MetS and Its Criteria

The prevalence of MetS was higher among patients in Tomsk (Table 10), compared with Kemerovo [*p* = 0.008; Odds Ratio (OR): 2.283, 95% Confidence Interval (CI): 1.233–4.228] or Omsk (*p* = 0.004; OR: 2.517, 95% CI: 1.333–4.754), mainly due to the high prevalence of abdominal obesity (Table 10), while men from Tomsk (Table 11) were more susceptible to this condition than men from other regions (*p* < 0.05 in each case). The incidence of elevated HDL-C levels was higher in individuals from Kemerovo than in individuals from Omsk (Table 10), mainly due to women (*p* < 0.05 in each case; Table 12). Blood pressure was higher in the female part of the study population in female patients from Omsk compared to female patients from Kemerovo (*p* = 0.01; Table 12). The number of MetS criteria was comparable in all the regions studied (Table 13); there were also no gender differences.

**Table 10:** Prevalence of MetS and its criteria in the study population [*n* (%)].

**Parameter**	**Psychiatric hospital patients**, ***n***			
	**(1) Kemerovo, 94**	**(2) Tomsk, 131**	**(3) Omsk, 91**	***p*-value**
MetS prevalence	19 (20.2%)	48 (36.6%)	17 (18.7%)	*p*_1−2_ = 0.008^*^
				*p*_1−3_ = 0.793
				*p*_2−3_ = 0.004^*^
Waist circumference	36 (38.3%)	76 (58.0%)	35 (38.5%)	*p*_1−2_ = 0.004^*^
				*p*_1−3_ = 1.000
				*p*_2−3_ = 0.006^*^
Blood pressure	19 (20.2%)	36 (27.5%)	25 (27.5%)	0.398
Fasting glucose	18 (19.1%)	19 (14.5%)	9 (9.9%)	0.203
Triglyceride	31 (33.0%)	46 (35.1%)	19 (20.9%)	0.062
HDL-C	69 (73.4%)	83 (63.4%)	50 (54.9%)	*p*_1−2_ = 0.112
				*p*_1−3_ = 0.009^*^
				*p*_2−3_ = 0.208

* *p < 0.05, statistically significant difference; these data were analyzed using the χ^2^ test. MetS, metabolic syndrome; BMI, body mass index; HDL-C, high-density lipoprotein cholesterol*.

**Table 11:** Prevalence of MetS and its criteria in the male part of the study population [*n* (%)].

**Parameter**	**Psychiatric hospital patients**, ***n***			
	**(1) Kemerovo, 48**	**(2) Tomsk, 69**	**(3) Omsk, 50**	***p*-value**
MetS prevalence	9 (18.8%)	26 (37.7%)	5 (10%)	*p*_1−2_ = 0.028^*^
				*p*_1−3_ = 0.216
				*p*_2−3_ = 0.001^*^
Waist circumference	13 (27.1%)	33 (47.8%)	9 (18.0%)	*p*_1−2_ = 0.024^*^
				*p*_1−3_ = 0.281
				*p*_2−3_ = 0.001^*^
Blood pressure	13 (27.1%)	21 (30.4%)	10 (20.0%)	0.439
Fasting glucose	7 (14.6%)	10 (14.5%)	5 (10.0%)	0.730
Triglyceride	16 (33.3%)	29 (42.0%)	13 (26.0%)	0.188
HDL-C	31 (64.6%)	47 (68.12)	25 (50.0%)	*p*_1−2_ = 0.327
				*p*_1−3_ = 0.314
				*p*_2−3_ = 0.042^*^

* *p < 0.05, statistically significant difference; these data were analyzed using the χ^2^ test. MetS, metabolic syndrome; BMI, body mass index; HDL-C, high-density lipoprotein cholesterol*.

**Table 12:** Prevalence of MetS and its criteria in the female part of the study population [*n* (%)].

**Parameter**	**Psychiatric hospital patients, n**			
	**(1) Kemerovo, 46**	**(2) Tomsk, 62**	**(3) Omsk, 41**	***p*-value**
MetS prevalence	10 (21.7%)	22 (35.5%)	12 (29.3%)	0.301
Waist circumference	23 (50.0%)	43 (69.4%)	26 (63.4%)	0.119
Blood pressure	6 (13.0%)	15 (24.2%)	15 (36.6%)	*p*_1−2_ = 0.148
				*p*_1−3_ = 0.01^*^
				*p*_2−3_ = 0.175
Fasting glucose	11 (23.9%)	9 (14.5%)	4 (9.8%)	0.181
Triglyceride	15 (32.6%)	17 (27.4%)	6 (14.6%)	0.143
HDL-C	38 (82.6%)	36 (58.1%)	25 (61.0%)	*p*_1−2_ = 0.07
				*p*_1−3_ = 0.024^*^
				*p*_2−3_ = 0.769

* *p < 0.05, statistically significant difference; these data were analyzed using the χ^2^ test. MetS, metabolic syndrome; BMI, body mass index; HDL-C, high-density lipoprotein cholesterol*.

**Table 13:** Number of MetS criteria in the study population [*n* (%)].

**Number of MetS criteria**	**Psychiatric hospital patients**, ***n***			
	**(1)—Kemerovo, 94**	**(2) Tomsk, 131**	**(3) Omsk, 91**	***p*-value**
0	10 (10.6%)	19 (14.5%)	23 (25.3%)	0.065
1	29 (30.9%)	35 (26.7%)	25 (27.5%)	
2	31 (33.0%)	27 (20.6%)	23 (25.3%)	
3	16 (17.0%)	30 (22.9%)	14 (15.4%)	
4	6 (6.4%)	18 (13.7%)	5 (5.5%)	
5	2 (2.1%)	2 (1.5%)	1 (1.1%)	

*These data were analyzed using the χ^2^ test; MetS, metabolic syndrome*.

### Determinants of MetS in Multiple Regression Analysis

The rest step in the statistical analysis was multiple regression to determine the potential impact of factors such as duration of disease, education level, employment status, city, PANSS score, BMI, and antipsychotic therapy. Chlorpromazine equivalents data were converted using a decimal logarithm to reduce asymmetry. MetS was associated with a duration of disease (*p* = 0.0026) and BMI (*p* < 0.0001) [adjusted *R*^2^ = 0.2435, *p* < 0.0001] as a result of the multiple regression model (Table 14).

**Table 14:** Determinants of MetS in multiple regression analysis.

**Variable**	***B***	***p*-value**
City	0.004981	0.8706
Education level	−0.018710	0.5107
Employment status	−0.030490	0.4076
Duration of disease	0.010260	0.0026^*^
Total antipsychotic dose (log_10_)	−0.037360	0.6241
Antipsychotics generation	0.0824486	0.1301
Number of antipsychotics	0.051550	0.2314
Antipsychotic therapy duration	−0.001682	0.6548
BMI	0.033480	<0.0001^*^
PANSS positive symptoms score	−0.000056	0.9896
PANSS negative symptoms score	0.000243	0.9517
PANSS total score	−0.001796	0.4006

* *p < 0.0001, adjusted R^2^ = 0.2435. BMI, body mass index; PANSS, positive and negative syndrome scale*.

## Discussion

### Employment Status

Patients from Tomsk were employed more often than the rest of the patient groups and had more frequent MetS compared to the Kemerovo subpopulation. This is consistent with Lin et al. (35) and Moreira et al. (36), that there are some differences in the higher prevalence of MetS in people with a lower socioeconomic level compared to a high one in the general population, which is apparently related to differences in diet and lifestyle. Also, Moreira et al. (36) pointed out that employment status should be considered in combination with other factors, in particular, the level of education and physical activity in the MetS development context. Patients from different hospitals included in our study had similar lifestyles and did not differ in educational level, which underlines the importance of the results of this part of the study.

### Schizophrenia Clinical Characteristics Comparison

It is known that the duration of schizophrenia increases the incidence of MetS in patients (5). This pattern was not reflected in the results of our study when comparing the duration of illness between patients from different hospitals and the presence of MetS in them. On the other hand, it is necessary to clarify that we found differences in the symptoms of schizophrenia between patients of different hospitals, which could affect the results obtained. Such differences were found in both males and females. The peculiarities described are more attributable to differences in study designs than to the contra data identified. For example, a higher incidence of obesity in chronic schizophrenia was noted in women (37), and according to Lin et al. (35) a higher prevalence of MetS is associated with older age, female sex, low psychotic symptoms, and clozapine use. In contrast, our study participants did not take clozapine, and the samples from different hospitals did not initially differ by sex.

### Comparison of Antipsychotic Therapy

Individuals diagnosed with schizophrenia often lead unhealthy lifestyles, such as being inactive, exercising little, eating poorly, and smoking a lot, which contributes to the MetS development. These are due in part to the negative symptoms of schizophrenia, lack of motivation, poor understanding of their health, and the sedative effects of antipsychotic treatment (38). Patients from Tomsk mostly received therapy with atypical antipsychotics, while patients from Omsk received conventional antipsychotics. The revealed differences in the prevalence of MetS among patients from Tomsk and Omsk may also be associated with antipsychotic therapy since it is well-known that the use of second-generation antipsychotics is more often accompanied by the development of metabolic disorders (27). We have previously shown that 6-week therapy with atypical antipsychotics led to a disruption of most parameters of fat metabolism, glucose, and lipid metabolism (39). Also, patients from Tomsk most often took antipsychotic monotherapy, patients from Omsk—a combination of two antipsychotics, and patients from Kemerovo were more likely to take three antipsychotics. Although the use of antipsychotic polypharmacy is controversial due to the possible increase in side effects, in routine clinical practice it often continues to be used in the treatment of patients with schizophrenia (28, 40). In our study, patients received a combination of conventional and atypical second-generation antipsychotics, excluding clozapine. A number of studies have shown conflicting data on the relationship between antipsychotic polypharmacy and MetS, with some of them demonstrating a possible increase in the risk of developing MetS, while others—its reduction (40, 41). Safe combinations of antipsychotic therapy in terms of the development of MetS are discussed when antipsychotics usually used in clinical practice are combined with a third-generation drug (aripiprazole). Presumably, this effect of aripiprazole may be associated with its partial agonist activity against the 5-HT1A serotonin receptor, which possibly balances the effects exerted through the 5-HT2C receptor, as well as with the effect on the orexin and histamine systems (42, 43). Also, it is known that antipsychotic polypharmacy does not increase the risk of MetS in schizophrenia patients when the total antipsychotic load in chlorpromazine equivalent is comparable to the equivalent dose in monotherapy (40). Our study also showed that polypharmacy does not play a significant role in the formation of MetS.

### Anthropometric Parameters

The highest prevalence of MetS, mainly due to an increase in the waist and BMI, was observed in patients from Tomsk, both in the general group and among males and females separately. This fits into the differences in approaches to antipsychotic therapy between hospitals discussed above and the general pattern of the formation of MetS, revealed in many studies, in particular, a large systematic review found that in patients with severe mental illness, a higher BMI was associated with lower levels of physical activity as a consequence of the lack of resources or professional support (44). Our previous study also showed the role of the bone component of body composition in the formation of MetS (45).

### Laboratory Parameters

BMI, HDL, triglycerides, waist circumference are all the strong risk factors for the development of MetS in drug naïve schizophrenia patients (46), and an unfavorable lipid profile is combined with increased BMI and waist circumference values (47). It is also known that women with chronic schizophrenia have more severe lipid metabolism dysfunction than men (37). We found differences in the level of fasting glucose in the blood serum between patients from Kemerovo and Omsk both in the general group and among males and females separately. The triglyceride level was higher in patients from Tomsk, mainly females than from Omsk. Females from Kemerovo had a lower HDL-C level than females from other regions. The data obtained ultimately influenced the prevalence of MetS and its components.

### Prevalence of MetS and Its Criteria

The prevalence of MetS was higher among patients in Tomsk, compared with other groups. Also, we found the sex differences in MetS criteria between groups. The minimum incidence of MetS was observed in patients from Omsk. At the same time, patients from Omsk had the highest severity of the disease according to PANSS, and patients from Tomsk had the lowest severity of positive symptoms according to PANSS. Other researchers previously showed that a deterioration in the lipid profile may be associated with an improvement in the clinical manifestations of schizophrenia (48). According to Lally et al. (49), an increase in triglyceride levels strongly predicted clinical improvement as well as an improvement in positive and negative PANSS scores. It is also known that people with schizophrenia had poor diets, including eating few fruits and vegetables, and a large number of instant foods, convenience foods, and sugar (50, 51). On this side, the lower prevalence of MetS in patients from Omsk can be explained by a rather meager diet due to economic conditions, since most patients from this group had a psychiatric disability. In addition, patients from Omsk were less likely to receive atypical antipsychotics. The number of MetS criteria was comparable in patients of all psychiatric hospitals.

### Determinants of MetS in Multiple Regression Analysis

Statistical analysis showed that MetS was associated with schizophrenia duration and BMI. As a result of constructing a multiple regression model, in which the value of the presence/absence of MetS acted as a dependent variable, there was a weak regression equation, that is, the studied predictors are weakly related to this variable. According to the results of other multivariate logistic regression analysis, the risk of developing MetS in people with mental disorders, is associated with different factors: in men, they include low educational level, high BMI, long duration of mental illness, concomitant chronic physical diseases and diagnosed bipolar disorder, while in women, aggravating factors include high BMI, marriage, and older age (52). The results obtained in our study indicate that the duration of schizophrenia, BMI, and the characteristics of antipsychotic therapy are not key factors in the development of MetS in patients with schizophrenia. It is assumed that there is a possible general genetic, endocrinological, and inflammatory predisposition to MetS and schizophrenia. In obedience to a systematic review (53), several genes are strongly associated with MetS in schizophrenia patients such as the fat mass and obesity associated gene (FTO), leptin and leptin receptor genes (LEP, LEPR), methylenetetrahydrofolate reductase (MTHFR) gene, and the serotonin receptor 2C gene (HTR2C). The regulation of homeostasis systems, including the hypothalamus-pituitary-adrenal system and the inflammatory response, is often disrupted in patients with schizophrenia. These pathophysiological imbalances are also associated with the development of MetS (54). Nevertheless, it would be unjustified to ignore clinical, anthropometric, and pharmacological factors when assessing the risk of MetS in patients with schizophrenia.

We did not find studies, even published in Russian, that even approximately allowed us to compare the prevalence of MetS in the general population of Kemerovo, Tomsk, and Omsk. Such data would be helpful to comprehend the results of our study more fully. At the same time, the following clinical implications proceed from it. First, if our study is replicated in other regions by comparing several hospitals and finding similar differences, it would raise questions about the influence of pharmacological traditions on the long-term effects of treatment. Second, there will be also a question of comparing the styles of use of antipsychotics by individual psychiatrists. Third, we once again draw attention in general to the problem of the safety of schizophrenia treatment.

## Conclusion

Taken together, we performed a comparison of the metabolic syndrome prevalence in patients with schizophrenia from three hospitals in one region. Based on the results above schizophrenia patients of different psychiatric hospitals within the same region had some differences in MetS and its components prevalence, schizophrenia clinical characteristics antipsychotic treatment peculiarities. Future studies are needed to comprehending the reasons for these differences in the context of treatment and rehabilitation approaches improving.

### Study Limitations

Our study had limitations such as crossover design, which did not allow us to establish the temporal relationship between schizophrenia and MetS, as well as the lack of reliable information about which antipsychotics patients received during the entire period of illness. In addition, we did not assess patients' lifestyle (e.g., physical activity, sedentary, diet, and smoking) in this study.

### Study Strengths

We consider the strengths of our study to be the assessment with standardized instruments, the collection of a large number of laboratory and anthropometric parameters, and the rigorous way of MetS diagnostic, are making the study reproducible.

## Data Availability Statement

The raw data supporting the conclusions of this article will be made available by the authors, without undue reservation.

## Ethics Statement

The studies involving human participants were reviewed and approved by Ethics Committee of Mental Health Research Institute of the Tomsk National Research Medical Center of the Russian Academy of Sciences. The patients/participants provided their written informed consent to participate in this study.

## Author Contributions

EK, AK, and SI: conceptualization. EK and SI: methodology and data curation. AK, IM, and IP: software. EK, IM, and AK: validation and writing—original draft preparation. IM, AG, and IP: formal analysis. IM, AG, AB, and VG: investigation. EK and AK: resources. EK, IM, AL, and VG: writing—review and editing. AS and SI: supervision. NB: project administration. AB: funding acquisition. All authors have read and agreed to the published version of the manuscript.

## Conflict of Interest

The authors declare that the research was conducted in the absence of any commercial or financial relationships that could be construed as a potential conflict of interest.
